# Unravelling the selective transport of Co^2+^ and Hg^2+^ ions through functionalized graphene nanostructures from aqueous nitrate solution: a molecular dynamics simulation study

**DOI:** 10.1039/d6ra00091f

**Published:** 2026-05-08

**Authors:** Drisya G. Chandran, Rima Biswas

**Affiliations:** a Process Simulation Research Group, School of Chemical Engineering, Vellore Institute of Technology Vellore Tamil Nadu 632014 India rima.biswas@vit.ac.in

## Abstract

Molecular dynamics (MD) simulations were employed to unravel the atomistic mechanisms responsible for the selective permeation of cobalt (Co^2+^) and mercury (Hg^2+^) ions through chemically functionalized nanoporous graphene (GRA) membranes. The computational framework consisted of nanoporous GRA membranes functionalized with electronegative fluorine (–F) and chlorine (–Cl) moieties and immersed in mixed aqueous nitrate environments. An external electric field applied along the membrane normal induced directed ionic migration across the pores. Detailed structural and dynamical analyses reveal that ion transport is dictated by a delicate balance among hydration free energy, ion-pore electrostatic interactions, and interfacial polarization effects. The F-functionalized nanoporous GRA membranes have been shown to promote enhanced ion transport when subjected to an external electric field. Notably, Co^2+^ ions exhibit preferential permeation through F-functionalized pores, whereas Hg^2+^ ions demonstrate higher permeation efficiency in Cl-functionalized pores. These findings provide a fundamental molecular-level understanding of how functional group chemistry and applied electric fields modulate ion selectivity and transport energetics in GRA-based membranes with tailored pore diameters, offering predictive insights for the rational design of next-generation nanofiltration and electroseparation systems.

## Abstract 2

Molecular dynamics (MD) simulations were used to investigate the atomistic mechanisms that govern the selective penetration of cobalt (Co^2+^) and mercury (Hg^2+^) ions across chemically functionalized nanoporous graphene (GRA) membranes.

## Graphical abstract

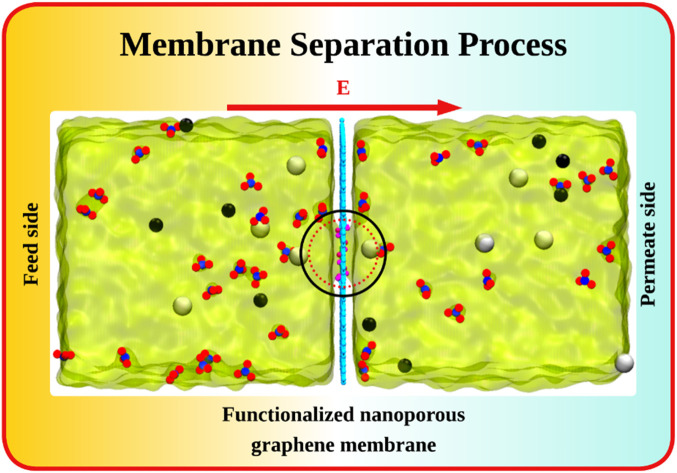

## 1 Introduction

The uncontrolled discharge of industrial wastewater containing heavy metals poses a significant environmental and public health risk worldwide.^1,2^ Industrial effluents often contain a wide range of toxic heavy metal contaminants, including mercury (Hg), cobalt (Co), copper (Cu), lead (Pb), cadmium (Cd), arsenic (As), chromium (Cr), and zinc (Zn).^3^ For instance, mercury is of particular concern due to its extreme toxicity and ability to cause severe physiological and neurological damage. Mercury exposure has been linked to immune system suppression, reproductive disorders, sensory and cognitive impairments, kidney dysfunction, and central nervous system degradation.^4^ Mercury exists in multiple chemical forms, including elemental mercury (Hg^0^), ionic mercury (Hg^+^, Hg^2+^), methylmercury (MeHg^+^), and ethylmercury (CH_3_CH_2_Hg^+^). Specifically, divalent mercury (Hg^2+^) is a widespread environmental contaminant that is mostly present in surface water.^5^ Even at very low concentrations, Hg^2+^ ions permanently harm living things.^6^ Mercury can be found in significant quantities in wastewater from sources like chloralkali production facilities, electronics and electrical manufacturing facilities, sulphide ore roasting operations, and the manufacturing of batteries, lamps, paints, and paper products.^7^ Moreover, cobalt-containing compounds are widely used in the petrochemical industry.^8^ The production of paints and batteries frequently uses both metal ions. At elevated concentrations, Co^2+^ ions exhibit toxic effects. Although it is less hazardous than mercury, prolonged or excessive exposure can still pose substantial risks, including neurotoxicological symptoms, partial or complete loss of smell, and dilation of the heart.^9^ According to the US EPA, the drinking water criterion for mercury and cobalt must not exceed 2 µg L^−1^, 100 µg L^−1^, respectively.^10^ Therefore, the removal of toxic and non-biodegradable Hg^2+^ and Co^2+^ ions from industrial effluent is crucial before being released into the environment.

Experimental approaches for heavy metal remediation encompass ion exchange, chemical precipitation, flocculation, adsorption, reverse osmosis, electrochemical treatment, and biological methods.^11–15^ Among these, membrane separation technology has a major impact on the treatment of industrial wastewater and the protection of water resources.^16^ An ideal membrane should possess high chemical stability, exceptional water flux, strong antifouling properties, precise ionic and molecular sieving capabilities, high scalability, and cost-effectiveness for industrial applications. Unfortunately, the trade-off between membrane permeability and selectivity has prevented conventional membranes from meeting the growing need for water purification. The membranes will lose their selectivity when ultrahigh permeability is pursued and *vice versa*. Consequently, to get around the trade-off effect, it is imperative to build innovative membranes with high permeability and selectivity. The thickness of the membrane and the amount of flux are inversely correlated.^17^ Thus, thin membranes are of great importance.

Nanotechnology's revolutionary breakthrough resulted in a range of nonporous membranes for water treatment applications.^18^ In recent years, two-dimensional (2D) materials such as graphene (GRA) have garnered a lot of attention as potential solutions to break the trade-off effect in water desalination and purification because of their ultrathin 2D structure, high specific surface area, tunable pore size, good chemical stability, excellent mechanical strength, and controlled physicochemical properties.^19–23^ In particular, functionalized and nanoporous GRA membranes offer unique characteristics and applications.^24–26^ A perfect GRA sheet is impermeable to molecules as tiny as helium because it has no holes.^27^ This is because atoms attempting to cross the membrane are repelled by the abundant electron density of its aromatic rings. Thus, permeability can only be achieved if the GRA sheet has drilled holes in it.

In recent times, several experimental investigations have been conducted to produce holes in GRA.^28–30^ Cavitary GRAs have been recognised as extremely effective membrane materials for the separation of ions, gases, and nanoparticles.^31,32^ Even though many research studies have shown that GRA has various uses in a variety of fields, including DNA sequencing, gas separation, and metal ion removal.^33–37^ The GRA membranes feature high water permeability and strong salt rejection, which have been examined as an effective method of water purification.^38^ All of these systems are engineered such that ions do not pass and water molecules penetrate *via* the nanopores, resulting in pure water. Research is still needed to fully understand the role and importance of nanoporous GRA structure for the selective permeation of heavy metal ions from aqueous solutions. Moreover, nitrate (NO_3_^−^) ions are common in various industries, and studying their behavior provides a broader understanding of membrane performance across different ionic environments. While most studies emphasize chloride-based systems, investigating nitrate-containing environments can uncover distinct ion–membrane interactions and transport behaviours, thereby providing a more comprehensive understanding of membrane selectivity and functionality.

However, to the best of our knowledge, there is no report available in the literature on the selective permeation of Hg^2+^ and Co^2+^ ions from the nitrate salt solution under the influence of an external electric field by functionalized nanoporous GRA using experimental or theoretical methods. The transport behaviour and mechanism of ions and water molecules *via* nanopores during the separation process are particularly confined mass transport and remain ambiguous. Thus, the researchers intended to address this research lacuna by implementing a functionalized ion-gated nanoporous structure. Therefore, in this research work, we performed classical molecular dynamics (MD) simulations to understand the unique structure, separation, and permeation properties of functionalized nanoporous GRA for Hg^2+^, Co^2+^ ions, and water molecules under the influence of an external electrical field. Furthermore, the concentration gradient across the membrane serves as a primary driving force for ion transport. At low ion concentrations, the gradient may not be sufficient to achieve effective separation, while at excessively high concentrations, intense competition for membrane binding sites can occur and which leads to reduced separation performance. Elevated ion concentrations may also destabilize the membrane structure over time, promoting degradation or fouling. Hence, this study specifically focuses on assessing the influence of the applied electric field and pore functionalization on selective ion transport. The insights obtained from this work may contribute to the design of energy-efficient functionalized nanoporous GRA membrane for the selective permeation of heavy metal ions in water purification systems. In addition, the recovery and reuse of Hg^2+^ and Co^2+^ ions in various technological and scientific applications represent valuable strategies for minimizing waste and conserving natural resources. Such efforts are aligned with global sustainability initiatives, particularly sustainable development goals (SDGs) including SDG 6 (*i.e.*, clean water and sanitation), SDG 12 (*i.e.*, responsible consumption and production), and SDG 14 (*i.e.*, life below water), which emphasize improving water quality, promoting responsible chemical management, and reducing pollution in aquatic environments.^39^ The subsequent sections outline the MD simulation approach and discuss the findings of our research.

## 2 Computational details

**Fig. 1:**
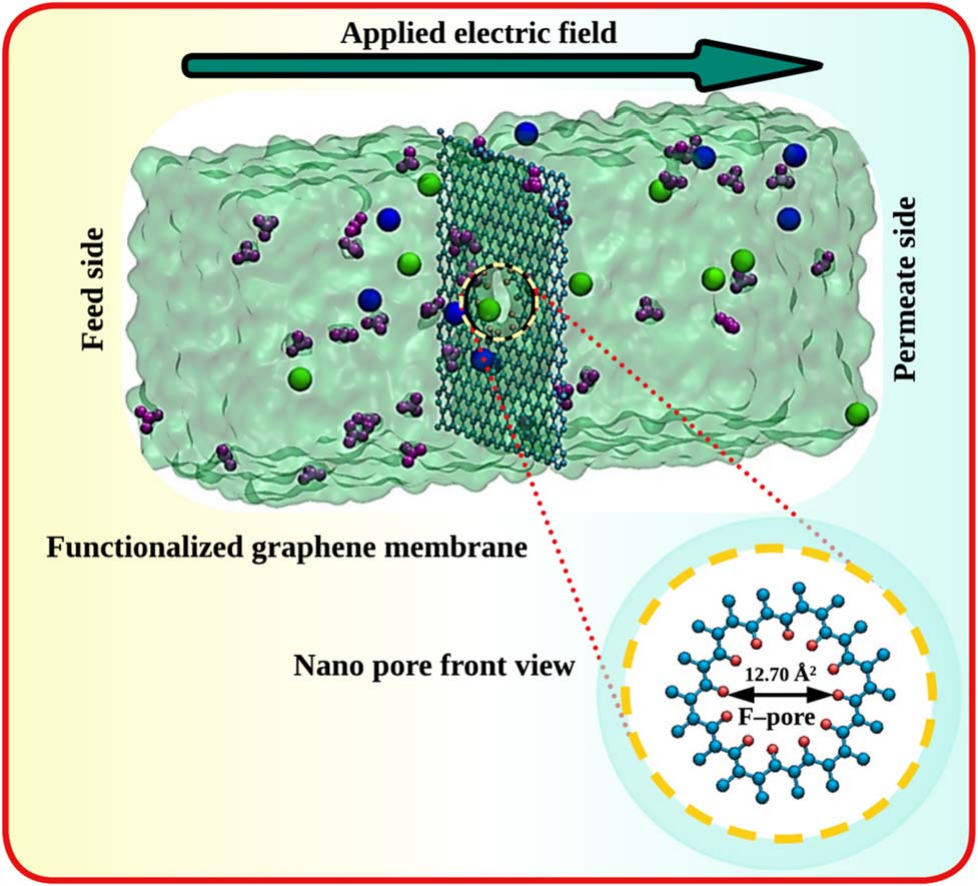
Snapshot of the simulated MD system. Carbon, fluorine, cobalt, mercury, and nitrate are represented by cyan, pink, green, blue, and purple colors respectively.

**Fig. 2:**
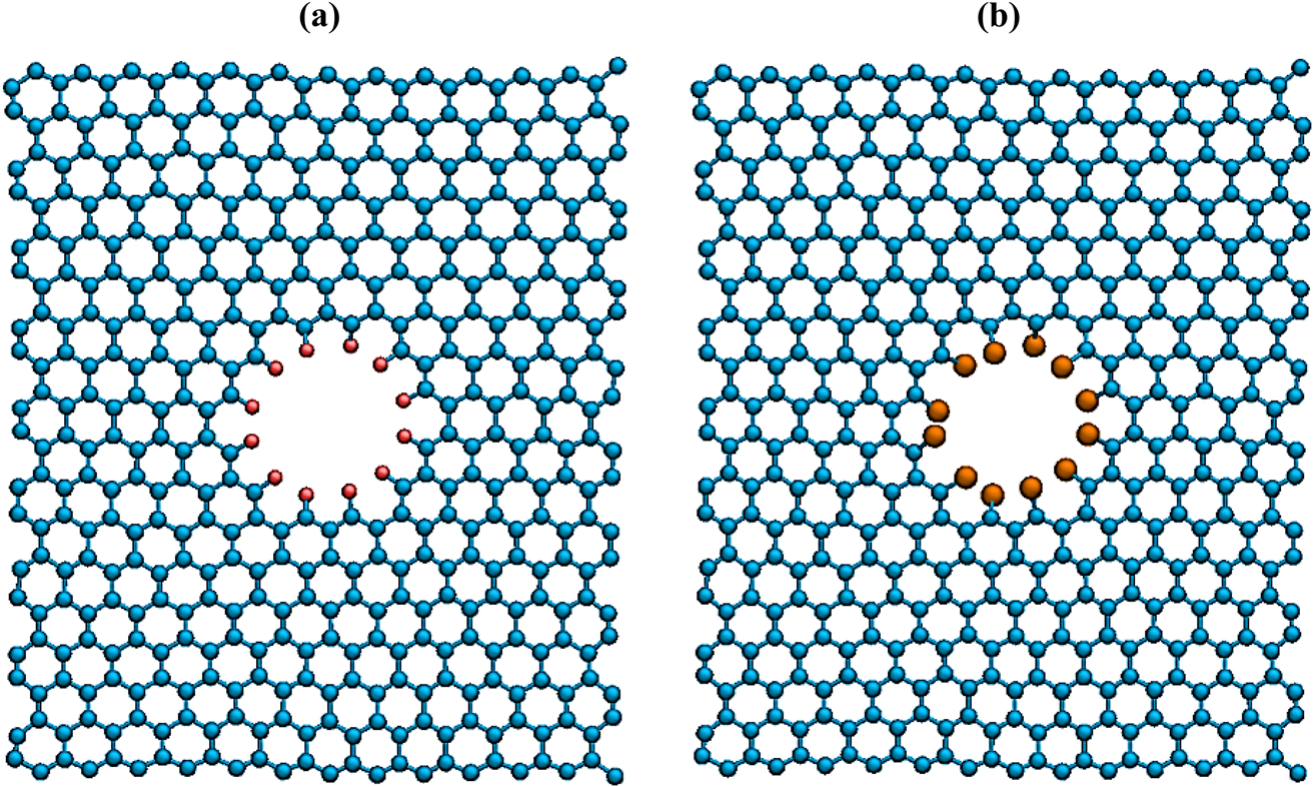
Snapshots of nanoporous GRA membrane functionalized with (a) fluorine, and (b) chlorine. Carbon, fluorine, and chlorine are represented by cyan, pink, and orange colors respectively.

All the MD simulations were performed with NAMD version 2.9.^40^ The systems were modeled with 3D periodic boundary conditions. The initial configuration for MD simulation was developed using the packmol program^41^ with the dimensions of 30 × 35 × 80 Å^3^. The sizes of the GRA were 30 × 35 Å^2^. A snapshot of the MD system was shown in Fig. 1. The molecular system consisted of functionalized nanoporous GRA immersed in a mixed nitrate salt solution (*i.e.*, Hg(NO_3_)_2_ and Co(NO_3_)_2_) containing metal ions and an appropriate number of water molecules. In real industrial wastewater, the concentration of dissolved heavy metal ions typically ranges from a few ppm to several hundred ppm, depending on the nature of the industrial effluent. In the present study, a concentration of 0.4 M was used^36,42,43^ because the simulation domain is confined to nanometer-scale dimensions and nanosecond timescales. At experimentally realistic concentrations, the number of ions present within the limited simulation box would be extremely small, often less than one ion on average, thereby preventing statistically meaningful observation of ion–membrane interactions and permeation events within the accessible simulation time. Although this concentration exceeds those typically found in real industrial effluents, the simulations are designed to capture the fundamental molecular-level interaction behavior between heavy metal ions and functionalized GRA membranes. Therefore, the resulting trends in ion selectivity and permeation can still provide qualitative insights relevant to experimentally realistic concentration regimes. The porous membrane was held to be fixed during the simulation. Initially, various chemical functional groups, including carboxylate anion (–COO^−^), fluorine (–F), sulfonic acid (–SO_3_H), chlorine (–Cl), and trifluoromethyl (–CF_3_) were evaluated for nanopore edge functionalization of GRA to enhance ion selectivity (see Fig. S1 and Table S1). However, some of these modifications proved ineffective due to unfavourable ion-pore interactions, excessive water flux, inadequate ion permeability, and structural incompatibility under simulation conditions. Therefore, to improve the selective permeation of heavy metal ions, GRA nanopores functionalized with fluorine (F-pore) and chlorine (Cl-pore) were selected for detailed investigation. Functionalized GRA pores were built by using the molecule visualization and editing program GaussView 5.0, while the geometry optimizations were carried out in the Gaussian 09 package using the B3LYP functional with 6-311G basis set and it is shown in Fig. 2. A nanopore was introduced at the center of the GRA membrane by constructing a sheet consisting of 444 carbon atoms. A smaller pore would sterically exclude ions by forcing extensive dehydration, resulting in prohibitively high energy barriers for transport, whereas larger pores would compromise selectivity by permitting the co-permeation of water and nitrate ions. In this study, a GRA membrane with two types of functionalized nanopores of different sizes was employed to separate heavy metal ions from aqueous solution. During model construction, the pore edge was saturated with 12 fluorine atoms to form the F-pore system, while 12 chlorine atoms were used to generate the Cl-pore configuration. These pores were designed to investigate the effect of pore-edge functionalization on selective ion permeation behavior. The resulting pore areas were 12.70 Å^2^ and 11.52 Å^2^ for the F-pore and Cl-pore systems, respectively. The pore diameter was calculated from the open pore area measurements using eqn (1). The carbon atoms of the GRA framework were assigned zero partial charges because pristine GRA consists of an extended sp^2^-hybridized π-conjugated network with highly delocalized electrons and no intrinsic permanent dipole moment. This modelling approach allows the electrostatic influence of the pore-edge functional groups to be isolated when analyzing ion permeation behavior.1
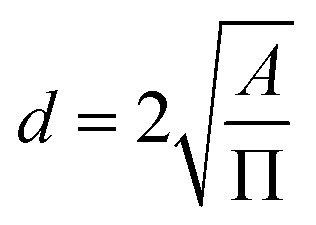


Classical MD simulations were carried out by using all-atom OPLS force field parameters^44^ due to its ability to accurately reproduce structural and transport properties of aqueous ionic systems.^45,46^ The non-bonding parameters were tabulated in Table 1. The TIP3P model was employed to represent water molecules in the present simulations owing to its compatibility with widely adopted force fields and favorable computational efficiency.^47^ Although the choice of water model can affect quantitative properties such as water flux and diffusion coefficients, the qualitative transport behavior and the fundamental mechanistic insights governing confined water and ion permeation remain largely unchanged.^48^ The parameters for GRA and F-GRA were taken from Kommu *et al.*,^38^ while Cl-GRA were obtained from LigParGen.^49–51^ The parameters for nitrate ions from Canongia Lopes *et al.*^52^ Long-range electrostatic interactions were calculated using the Particle Mesh Ewald summation method. The total charge of the molecular assembly was neutralized for all the simulations by introducing an appropriate number of counter ions. Ensuring overall electroneutrality is essential when employing long-range electrostatic interactions under periodic boundary conditions, as non-neutral systems may introduce spurious electrostatic artifacts and lead to non-physical force and energy contributions.^53^ The interaction between GRA–water and GRA–ion was described by using Lennard-Jones (LJ) and coulombic potentials. The bonded interactions in the NO_3_^−^ ions are described by using harmonic potentials.^54,55^ The non-bonded interactions were calculated using a 12 Å atom-based cut-off. An electric field was applied perpendicular to the nanoporous GRA membrane during the simulation (see eqn (2)), and (3) was used to calculate the current. Here, *E*, *V*, *l*_*z*_, *I*, *n*, *q*, Δ*t* are the external electric field (kcal mol^−1^ Å^−1^ e^−1^), potential difference (volt), and system size along the *z*-axis (Å), current (pA), average number of ions passing the GRA membrane, ion charge, and simulation time (ns), respectively.^36,56^2
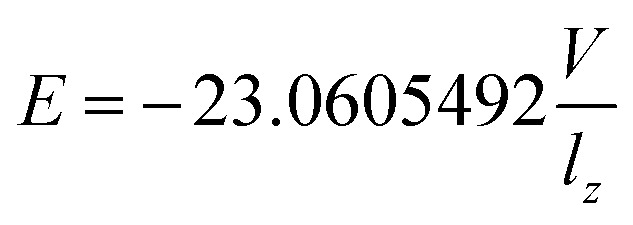
3
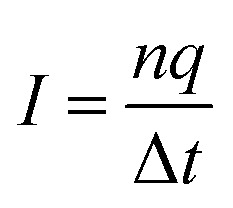


**Table 1:** List of non-bonding parameters

Molecules/ions	Site	Charge (*e*)	*σ* (Å)	*ε* (kcal mol^−1^)
Water	O	−0.8340	3.1507	0.1521
	H	0.4170	0.0000	0.0000
GRA	C	0.0000	3.4000	0.0556
F–GRA	C	0.2200	3.4000	0.0690
	F	−0.2200	2.8500	0.0610
Cl–GRA	C	−0.0300	3.5418	0.0700
	Cl	−0.0664	3.3921	0.3000
–NO_3_^−^	N	0.9500	3.2070	0.1600
	O	−0.6500	3.3490	0.1700
Metal ion	Hg^2+^	2.0000	2.3690	0.0410
	Co^2+^	2.0000	2.1000	0.1811

The movement of molecules across a charged membrane brought on by an externally applied electric field is known as electroosmotic flow (EOF).^42^ In this investigation, EOF is often preferred over pressure-driven flow (PDF). Unlike PDFs, where velocity scales inversely with the square of the capillary radius, and pressure must be maintained to sustain flow, the voltage required for EOF is independent of the capillary radius. Membrane fouling, arising from the deposition of organic matter, biofilms, and dissolved salts, remains a major obstacle to efficient filtration, leading to concentration polarization, pore blockage, and reduced performance. The EOF membranes functionalized with negatively charged groups effectively suppress fouling through electrostatic repulsion and uniform ion transport under an applied electric field. The resulting electrophoretic forces assist in foulant removal, imparting a self-cleaning capability and enhancing membrane stability. Thus, EOF provides an energy-efficient and durable approach with improved ion selectivity and fouling resistance for advanced water purification applications.

The system must first be configured in its basic state. Thereafter, the system was subjected to a series of pre-processing stages involving 10 000 steps of energy minimization followed by slow and steady heating to reach 298 K using temperature reassigning parameters of NAMD. Then, an equilibration phase is performed for 5 ns at the NPT ensemble, during which the system evolves from its basic configuration. The structural thermodynamic parameters were checked throughout the equilibration process until stability was reached. The temperature was maintained by a Langevin thermostat, and pressure was monitored by using a Langevin piston at 298 K and 1 bar. Finally, with the optimized cell volume, the production run of 50 ns was performed at the NVT ensemble. The SHAKE algorithm was used to constrain the bonds connected with hydrogen. The Verlet algorithm was used to integrate the equations of motion with a time step of 1 fs. During the simulations, ions and water molecules were free to flow in all directions. The trajectories were collected and are used for further calculations. Typical snapshots were taken using VMD software.^57^

The coordination number (CN) was calculated by integrating *r*^2^*g*(*r*) up to the first minimum of the pair correlation function in eqn (4).^58–60^ Here, *r*_0_ and *r*_1_ denote the rightmost positions starting from *r* = 0 and the first minimum, respectively. The *g*(*r*) is approximately zero.4
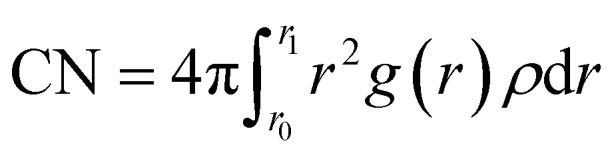


The self-diffusivity was calculated by using the Einstein equation shown in eqn (5).^61^ These values provide more quantitative insights into the mass transfer characteristics. Here, *r*_*i*_(*t*) and *r*_*i*_(0) are the positions of the *i*th atom at time instant *t* and at time instant 0, respectively. The ensemble block average of the squared displacements on the right-hand side of eqn (5) indicates the mean square displacement (MSD).

5 6*Dt* = 〈|*r*_*i*_(*t*) − *r*_*i*_(0)|^2^〉

## 3 Results and discussion

The permeation behavior of Hg^2+^ and Co^2+^ ions, *via* two distinct functionalized GRA nanopores, was systematically investigated using classical MD simulations, which offer an effective approach to ion transport research at the atomic level. Prior to applying an external electric field, a 50 ns MD simulation was performed to assess intrinsic transport behavior. Under these conditions, neither cations nor anions permeated through the functionalized pores. At the initial stage of the simulation, contaminated water containing a mixed metal ion solution was positioned on the feed side of the functionalized GRA membrane, while an equivalent amount of pure water was distributed on the permeate side to maintain hydrostatic equilibrium and eliminate pressure-driven artifacts. This arrangement made sure that any net movement of water molecules from the feed side to the permeate side that was seen during the simulation could only be ascribed to the force that was supplied externally and not to the initial conditions. A directed migration of ions toward the membrane was then induced by an external electric field with the goal of purifying the feed side by eliminating toxic heavy metal ions and moving them to the permeate side. Three different field strengths, such as 5 kcal mol^−1^ Å^−1^ e^−1^, 20 kcal mol^−1^ Å^−1^ e^−1^, and 35 kcal mol^−1^ Å^−1^ e^−1^, were applied to evaluate the field-dependent transport characteristics of the ions. Although the microscopic electric field strengths employed in these MD simulations are higher than the macroscopic fields typically used in industrial membrane systems, such field intensities are required to accelerate barrier-limited ion permeation within computationally accessible simulation timescales. This approach enables the observation of rare translocation events while preserving the fundamental nanoscale transport mechanisms governing ion selectivity and permeation through nanoporous GRA membranes.^36^ Comparable electric field magnitudes have been adopted in previous MD investigations^36,52^ of ion transport across functionalized nanoporous membranes to obtain statistically meaningful transport characteristics. The findings of the MD simulation revealed that Co^2+^ and Hg^2+^ ions were able to flow through the functionalized GRA pore under the simultaneous influence of applied field, pore diameter, and the proper nanopore functionalization. The number of heavy metal ions carried across the membrane pore is the main determinant of the efficacy of the removal processes in this case, not the flow of water across the membrane.

### 3.1 Density profile

**Fig. 3:**
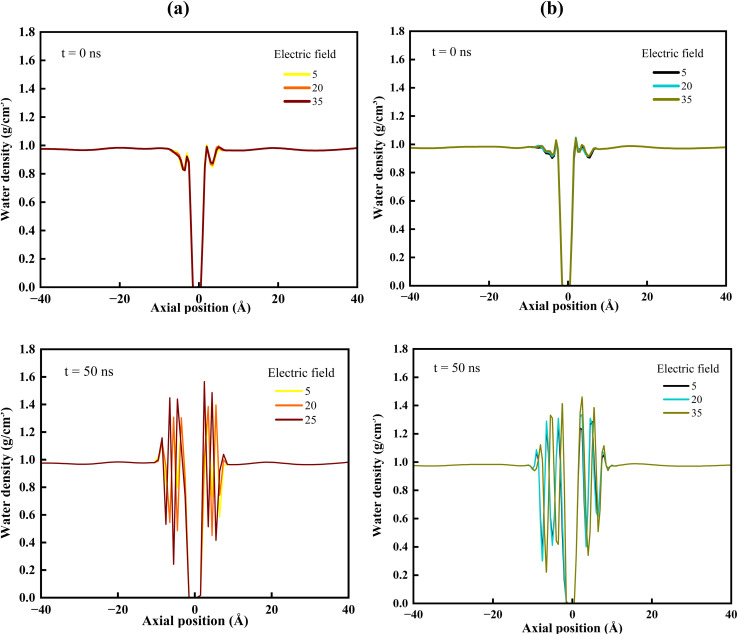
Density profile of water molecules near the (a) F-pore, and (b) Cl-pore GRA membrane system before and after the simulation w. r. t the *z* direction of the mixed system under the influence of the electric field.

We computed the initial and final water density profiles to examine the structural evolution of water within the nanoporous membrane separation system. The profiles clearly demonstrate that both the applied electric field and membrane properties, including surface and pore chemistry, significantly influence the spatial distribution and organization of water molecules across the membrane. As shown in Fig. 3, the mixed ion system exhibits distinct interfacial water structuring near the two differently functionalized nanoporous GRA membranes. That differ markedly from the uniform behaviour observed in the bulk region. Compared to the initial configuration, the water density profiles display well-defined layering and enhanced density modulation on both sides of the membrane at 50 ns. This is owing to the fact that the extended sp^2^-hybridized carbon network of GRA can induce layering of water and ions near the solid surface due to confinement effects and interfacial interactions between the fluid molecules and the hydrophobic GRA membrane, leading to an ordered arrangement parallel to the surface. Such interfacial layering has been widely reported for fluids near solid surfaces in molecular simulations.^62,63^ However, the presence of a nanopore functionalized with electronegative pore-edge functional groups introduces additional geometric and energetic effects due to localized electrostatic interactions that further influence the nearby ion and water accumulation.^64^ This accumulation results in a pronounced density peak in the vicinity of the pore, which is clearly visible from both F-pore (Fig. 3a) and Cl-pore (Fig. 3b) systems.

Despite this pronounced structuring near the membrane, the water density far from the interface remains close to the bulk value of ∼0.98 g cm^−3^, confirming that the perturbation is highly localized.^36^ Under an external electric field, water molecules exhibit partial orientational alignment and reorganize around the charged ions near the pore entrance, enhancing their accumulation on the feed side. Water flow is higher through the F-functionalized pore than through the Cl-functionalized pore due to its larger effective pore diameter (*i.e.*, ∼8.09 Å) compared to the Cl-pore (*i.e.*, ∼7.34 Å), which reduces steric resistance and facilitates water transport. In addition, the higher electronegativity of fluorine creates stronger localized electrostatic interactions at the pore edge, aligning nearby water dipoles under the applied electric field and enhancing electroosmotic coupling with migrating ions. As ions pass through the pore, they drag their hydration water molecules and thereby increasing water flux (Fig. 3a). In contrast, the larger atomic size of chlorine reduces the effective open area and introduces greater steric hindrance, thereby suppressing water permeation (Fig. 3b). Moreover, increasing the electric field strength further enhances water permeation. Overall, the water density profiles reveal that the interplay between electric field strength and functionalized nanoporous GRA membrane governs both the localization and mobility of water molecules, emphasizing the combined roles of interfacial structuring, ion hydration, and field-driven transport in determining water behaviour in the nanoporous GRA membrane system.

**Fig. 4:**
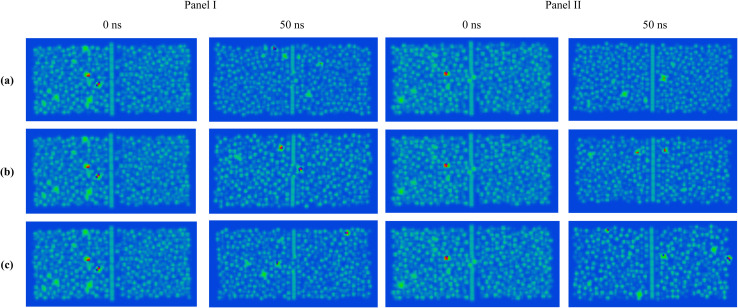
Density map snapshots of the F-pore (Panel I) and Cl-pore (Panel II) molecular systems containing heavy metal ions under three different external electric fields, including (a) 5 kcal mol^−1^ Å^−1^ e^−1^, (b) 20 kcal mol^−1^ Å^−1^ e^−1^, and (c) 35 kcal mol^−1^ Å^−1^ e^−1^, respectively.

The nanopore acts as the primary transport pathway for ions and water molecules. To examine spatial heterogeneity and pore-specific effects in the membrane system, density maps were constructed from the MD simulation trajectories. This approach enables high-resolution visualization of the local distribution of molecular species under an external electric field. As shown in Fig. 4a–c, the density maps reveal localized variations in molecular occupancy and highlight preferential transport pathways through the nanopore. In these maps, red regions indicate the highest probability of finding heavy metal ion presence, while blue regions represent the lowest probability.^65^

### 3.2 Metal ionic structure in the mixed system

**Fig. 5:**
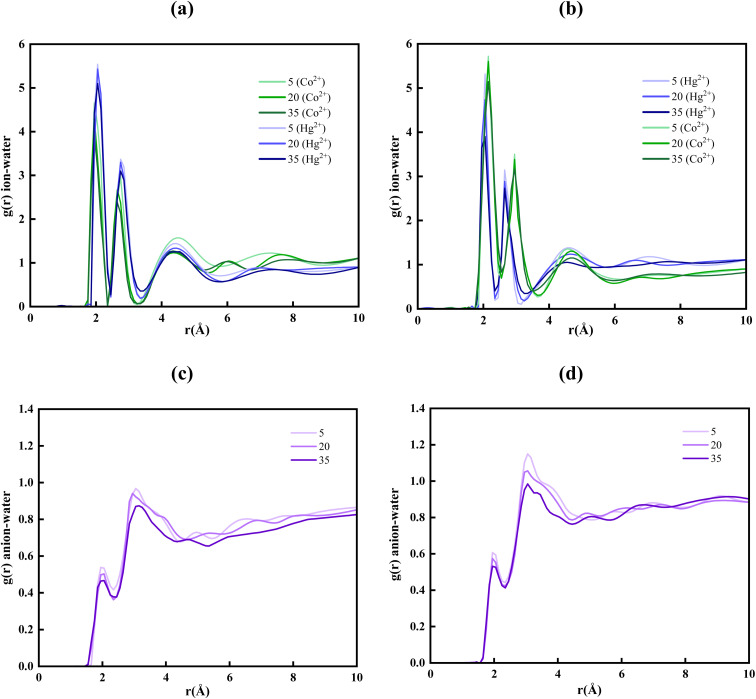
RDF of Co^2+^–water and Hg^2+^–water in the modelled (a) F-pore and (b) Cl-pore system under different external electrical fields. RDF of NO_3_^−^–water in the modelled (c) F-pore and (d) Cl-pore system under different external electrical fields.

To elucidate the field-dependent restructuring of the ionic hydration environment in the vicinity of the pore, the researchers estimated the radial distribution functions (RDFs) between the metal ions and water molecules in the mixed ionic system under three different external electrical field strengths. This pairwise correlation provides critical insight into the spatial organization and coordination behavior of solvent molecules surrounding the ions, thereby reflecting changes in the local solvation environment induced by the applied field. As reported by Impey *et al.*,^59^ metal ions in aqueous media are typically surrounded by a well-defined hydration shell composed of orientationally ordered water molecules governed by strong electrostatic interactions. Within confined geometries such as nanopores, ion translocation is often accompanied by the co-transport of these hydration water molecules. The ensuing water flow seen during the simulations can thus be attributed to the coupled motion of ions and their hydration shells, where the externally imposed electric field perturbs the local chemical potential and drives collective ion–water displacement across the nanopore interface. Fig. 5 depict the RDFs of metal ion–water pairs, including Hg^2+^–water and Co^2+^–water within the F-pore (see Fig. 5a) and Cl-pore (see Fig. 5b) systems, respectively. These ion–water systems show how the intermolecular distances between water molecules and heavy metal ions modulate the microscopic structure of the aqueous environment. In both systems, pronounced first coordination peaks are observed, which indicates that water molecules preferentially accumulate in a spherical coordination shell around Hg^2+^ and Co^2+^ ions. This structural ordering arises predominantly from the strong electrostatic interactions between the positively charged metal centers and the partial negative charge on the oxygen atoms of water molecules. The resulting ion–dipole interactions stabilize distinct hydration structures for each heavy metal ion.

Furthermore, at particular distances from the metal ions, the RDF curves of Hg^2+^–water and Co^2+^–water exhibit prominent peaks. These prominent peaks correspond to regions of enhanced local density, indicating a higher probability of locating water molecules in the immediate coordination sphere of the metal ions. In the F-pore system, the first hydration shells appear at approximately 2.050 Å for Hg^2+^ and 1.950 Å for Co^2+^, while in the Cl-pore system, they shift slightly to 2.038 Å and 2.146 Å, respectively. These subtle shifts in the peak positions indicate that the local solvation environment of the ions is sensitive to the chemical nature of the pore rim. Although these shifts are small, they are physically meaningful and arise from the distinct local electrostatic and polarizable environments created by the F and Cl atoms at the pore rim. Additionally, the positions of the first maxima and minima corresponding to the primary and secondary hydration shells remain unchanged across increasing electric field strengths, suggesting that the electric field has no effect on the spatial location of hydration shells. However, a noticeable reduction in RDF peak magnitude is observed with increasing field intensity, signifying enhanced dynamical exchange and partial disruption of the hydration structure under stronger electrostatic driving forces. The present findings align well with previously published reports,^36,66,67^ further validating the observed ion–surface interaction behavior.

The NO_3_^−^–water RDFs further elucidate the hydration behaviour within different pore environments. The F-pore shows a comparatively reduced RDF peak, suggesting partial disruption of the anion hydration shell (Fig. 5c). This indicates a greater tendency toward dehydration in the F-pore. Overall, the RDF analysis confirms that stronger NO_3_^−^–water interactions in the Cl-pore enhance anion rejection (Fig. 5d).

### 3.3 Dehydration effect

According to eqn (4), the coordination numbers (CNs) of water molecules were quantified in the vicinity of the nanopore under varying electric field strengths to further substantiate the RDF findings. Because the actual separation process and the associated structural changes primarily occur near the pore entrance and within the pore region. The selection of molecules for CN analysis was performed based on their spatial position relative to the membrane along the *z*-axis. Therefore, analyzing the local coordination environment in this region provides more relevant information regarding the interaction and transport mechanisms. For Co^2+^ and Hg^2+^ ions, the position and intensity of the RDF peaks observed from Fig. 5 are distinct for each metal ion, indicating variations in their respective hydration structures and coordination numbers. These variations highlight the ion-specific solvation characteristics.

**Fig. 6:**
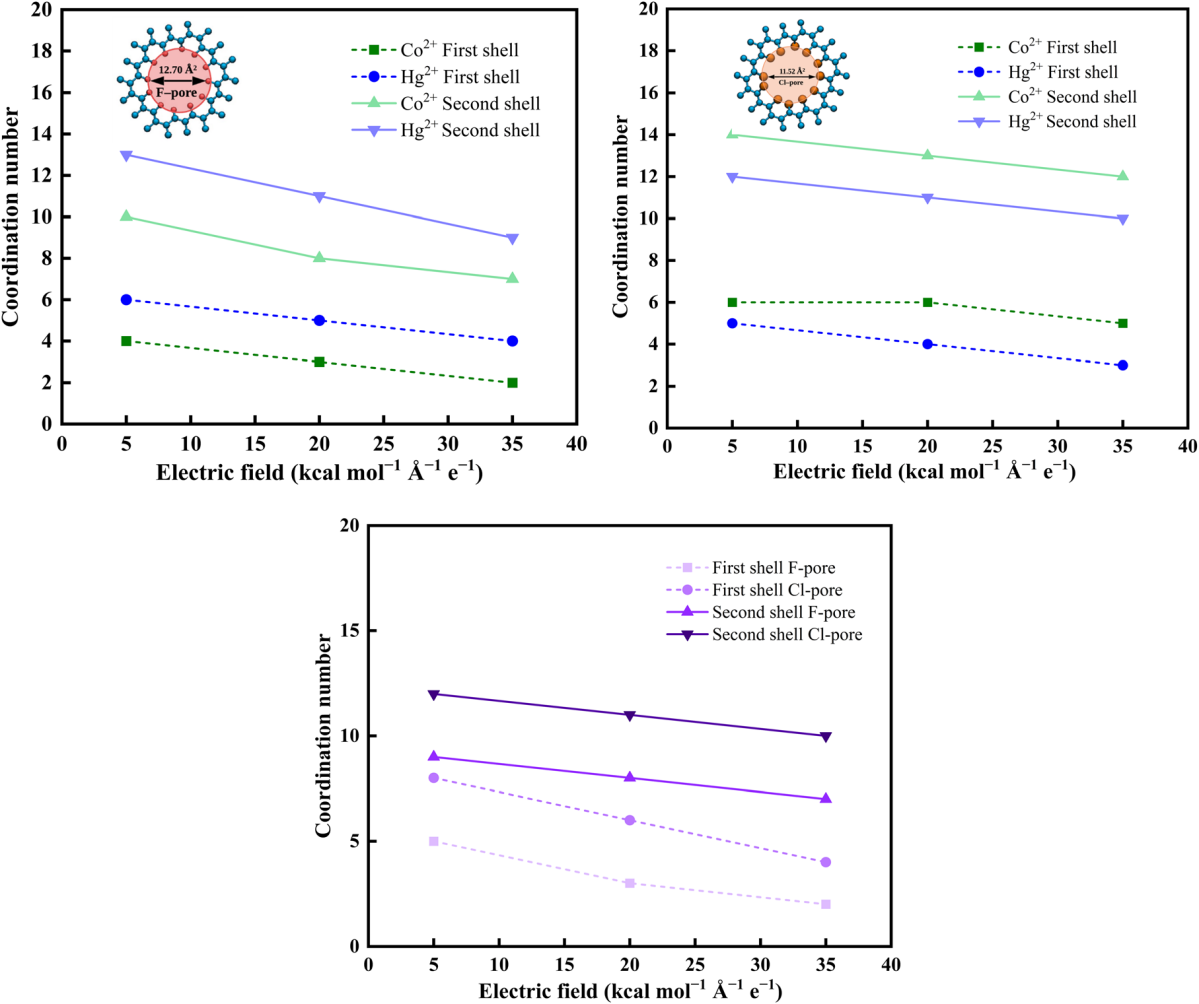
Electric field-induced dehydration of heavy metal ions in the (a) F-pore, and (b) Cl-pore system. (c) Dehydration of anions in the F-pore, and Cl-pore system.

Under the applied electric field, the water coordination numbers of both Co^2+^ and Hg^2+^ ions were found to decrease, indicating field-assisted dehydration during the permeation process. This behavior suggests that the increasing field strength facilitates the displacement of water molecules from the ions hydration shell, thereby lowering the energetic barrier for ion translocation through the functionalized nanopore. The coordination data clearly demonstrate that dehydration is strongly governed by pore chemistry and electric-field intensity. As observed from Fig. 6, the extent of hydration loss was more pronounced in the F-pore system (see Fig. 6a) than in the Cl-pore (Fig. 6b) system, owing to the higher electronegativity and local field intensity around the F atoms, which strongly perturb the hydration structure of the approaching ions and that leads to a systematic reduction in both first and second shell hydration numbers for Co^2+^ and Hg^2+^. On the other hand, the dehydration behaviour of NO_3_^−^ ions in Cl-functionalized GRA is noteworthy. The dehydration behaviour shown in Fig. 6c indicates that NO_3_^−^ retains a relatively stable solvation shell, which contributes to its highest rejection under various applied electric fields.

### 3.4 Ion permeation behaviour

**Fig. 7:**
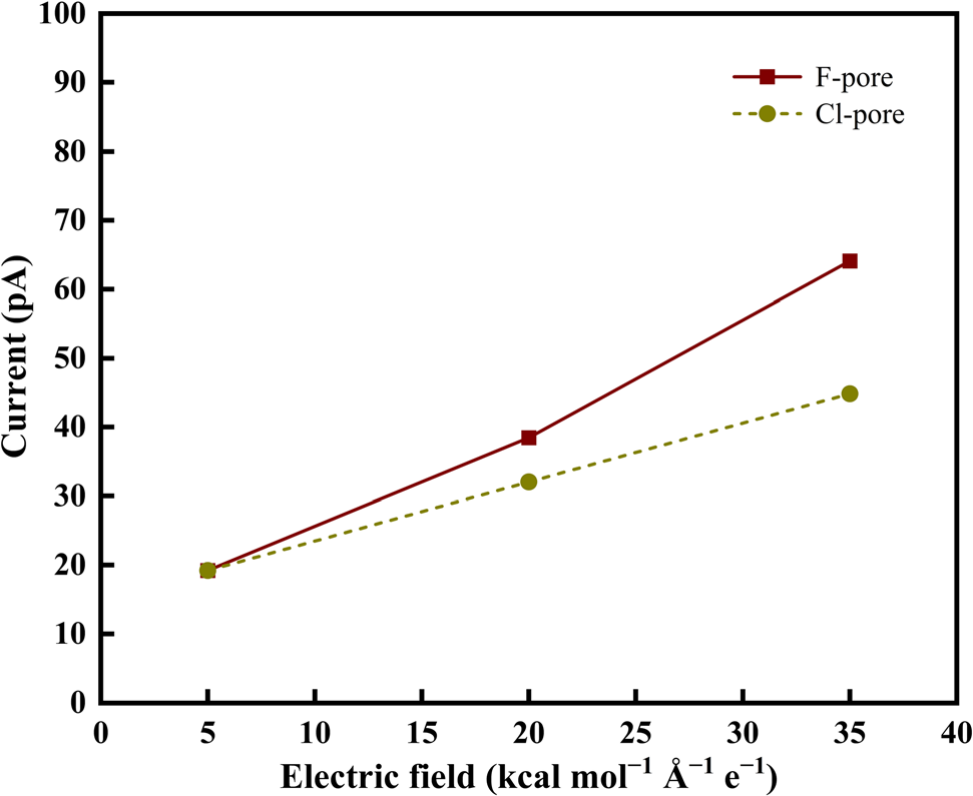
Current–electrical field curves for overall metal ion permeation across two distinct nanoporous membrane systems.

The total ionic current associated with the permeation of Co^2+^ and Hg^2+^ ions was evaluated for all applied electric field strengths using eqn (3). The resulting current–field profiles are depicted in Fig. 7 and clearly demonstrate that the applied electric field is directly proportional to the produced current. This trend arises because a stronger electric field exerts a greater electrostatic driving force on the ions, enhancing their drift velocity and thereby increasing the number of ions translocating through the nanopore per unit time. Thus, the escalation of current with increasing field strength directly reflects the enhanced ion transport efficiency induced by stronger electrical driving forces. Ions permeation through the GRA nanopores depends on the size of the pores, the functional groups that were utilized to chemically modify the pores, and the electric field that was applied.^36^

Effective translocation additionally depends on the ion's ability to dynamically reorganize its hydration shell as it approaches, traverses, and exits the nanoscale constriction. Several MD simulations have reported that the complete permeation of heavy metal ions from a chloride solution, even at lower field strengths.^36,68,69^ However, the current study examines the permeation behaviour of heavy metal ions in aqueous nitrate environments, focusing on the selective transport performance of F-pore and Cl-pore. This distinction is crucial, as NO_3_^−^ exhibits different hydration and interaction characteristics from chloride, thereby altering the permeation landscape and offering new insights into chemical-functionalization-driven selectivity. Under an external electric field, both ions are driven toward the pore in the same direction; however, their interactions with the pore rim differ significantly as a result of differences in effective pore size and the strength of ion-pore interactions.^70^ In the F-GRA system, the larger effective pore area reduces steric hindrance, thereby facilitating ion transport. As the ions approach the F-functionalized pore, Co^2+^ experiences a stronger electrostatic attraction to the highly electronegative F sites than Hg^2+^ due to its higher charge density and favourable hard–hard interaction characteristics. These interactions partially compensate for the dehydration energy required for translocation, thereby lowering the free energy barrier.^71,72^ Consequently, Co^2+^ exhibits enhanced sensitivity to the localized negative charges at the pore rim, promoting more effective access to and interaction with the pore entrance. This behavior is evident from Fig. 6 and 7. The larger size of Cl atoms reduces the effective pore diameter in the Cl-pore environment, leading to increased steric confinement. Moreover, the hydration shell of Co^2+^ remains largely intact due to the relatively weak electrostatic confinement imposed by the pore compared to the F-functionalized system. As a result, Co^2+^ retains a larger effective hydrated size, which hinders its entry into the smaller Cl-functionalized pore. Consequently, the permeation of Co^2+^ is reduced in the Cl-functionalized system. In contrast, the higher polarizability of Hg^2+^ enhances its induced interactions with the Cl-functionalized pore edges, thereby facilitating its permeation despite the steric constraints.^73^

**Fig. 8:**
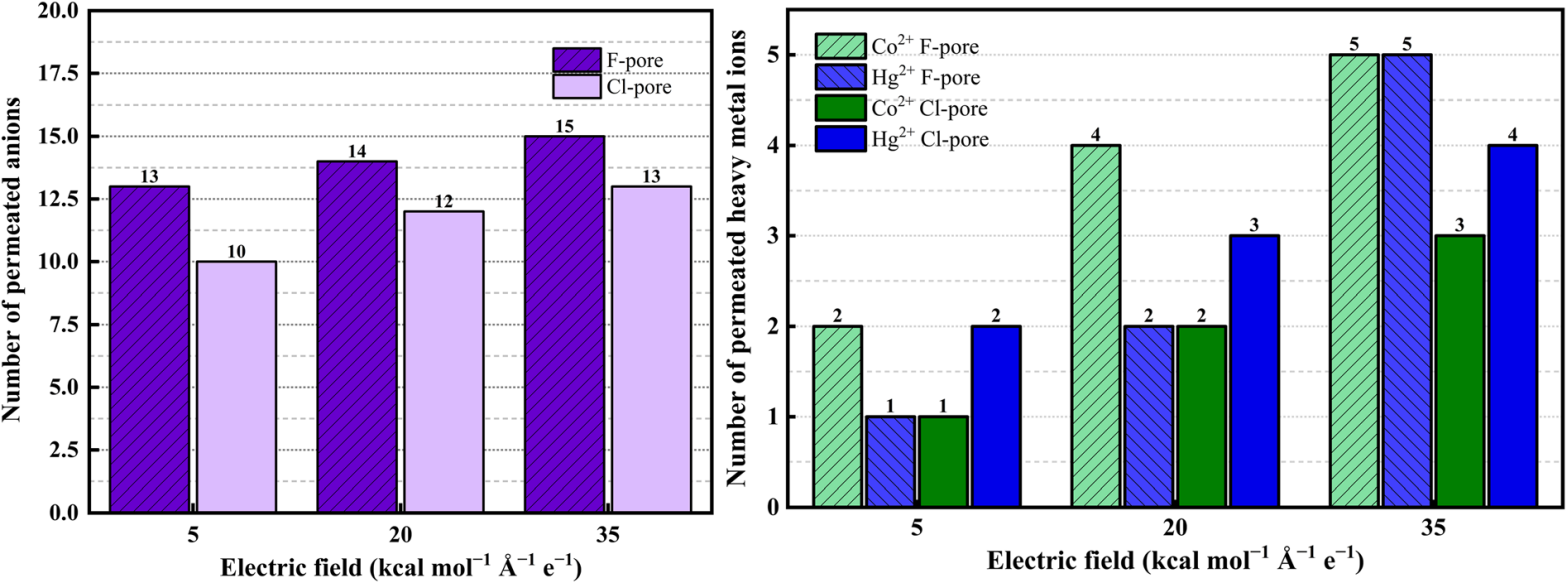
Number of (a) heavy metal ions and (b) NO_3_^−^ ions permeating through two nanoporous membrane systems.

On the other hand, the increased solvation and complexation of Hg^2+^ and Co^2+^ ions with NO_3_^−^ ions in aqueous media necessitate a higher external electric field for their effective permeation from nitrate-containing feed solutions. This behavior arises from the planar geometry of the NO_3_^−^ ion, its delocalized negative charge distribution, and its ability to form multiple hydrogen bonds with the surrounding water molecules. These features expand the effective hydrated size of NO_3_^−^ and promote a more structured solvent environment. The resulting enhancement in local ordering increases the solution viscosity, thereby reducing molecular mobility and lowering the diffusion rates of Hg^2+^ and Co^2+^ ions in the mixed ion system. Moreover, a clear competition in ion permeation was observed, governed by the specific chemical functionality present at the nanopore rim. At lower electric field strength (*i.e.*, 5 kcal mol^−1^ Å^−1^ e^−1^), ions encounter high resistance and mostly stay on the feed side. However, with increasing field strength (*i.e.*, 35 kcal mol^−1^ Å^−1^ e^−1^), ion migration across the nanoporous GRA membrane becomes more pronounced, resulting in their increased presence on the permeate side, as illustrated in Fig. 8a. All simulated systems showed the same field-dependent migration trend, underscoring the vital function that the electric field contributes in accelerating ion movement out of the aqueous nitrate solution.

Even though the primary intent of our study was to selectively allow Hg^2+^ and Co^2+^ ions to pass through the functionalized nanoporous GRA membrane while preventing NO_3_^−^ ions from doing so under an external electric field. As the applied electric field increases, the minimum number of NO_3_^−^ ions permeation was also observed at the end of 50 ns (Fig. 8b).

This limited passage arises because strong electric fields can drag counter ions toward the permeate side along with cations, particularly if they form ion pairs or transient complexes with cations. Furthermore, local concentration gradients and thermal variations close to the pore opening may occasionally enable NO_3_^−^ ions to cross the energy barrier. Thus, complete exclusion of NO_3_^−^ becomes challenging under strong driving forces. Nevertheless, the overall ion permeation observed in this study is lower compared to previously reported studies.^36,68,69^ This disparity can be explained by the different ionic environment of the metal nitrate solutions used here as opposed to the more widely used metal chloride systems.

### 3.5 Hydrogen bonding

The average number of hydrogen bonds formed between water molecules and the functionalized atoms at the nanopore rim (Table 2) exerts a decisive influence on solvent and ion transport across the functionalized GRA nanopores. Throughout the simulations, water molecules interacting with the pore edges continuously formed and dissociated hydrogen bonds, with the extent and stability of these interactions dictated primarily by the chemical nature of the functional groups.^74^ In the F-pore system, the high electronegativity of F promotes strong water–surface hydrogen bonding, particularly under low electric field strength (*i.e.*, 5 kcal mol^−1^ Å^−1^ e^−1^), where interfacial water molecules orient their hydrogen atoms toward the fluorinated rim, forming a stable and rigid hydrogen-bonding network. This structured interfacial network anchors water molecules near the pore entrance, suppresses their mobility, and reduces net water flux during the permeation of solvated Co^2+^ and Hg^2+^ ions, which is in line with the density profile of water molecules shown in Fig. 3a. As the electric field increases (*i.e.*, 20–35 kcal mol^−1^ Å^−1^ e^−1^), the field-driven migration of ions perturbs the local electrostatic environment and disrupts this hydrogen-bond network, progressively displacing interfacial water molecules from the pore vicinity. The highest average hydrogen bond formed between water molecules and F atoms was found to be 1.38 for the Co^2+^ ion and 1.62 for the Hg^2+^ ion, respectively. Despite the general reduction in hydrogen bonding at elevated fields, the Hg^2+^ ions consistently maintain a higher number of water–F hydrogen bonds than the Co^2+^ ions. This behavior arises from the stronger polarization induced by Hg^2+^, which intensifies the local electric field and enhances the orientationally ordering of water in the vicinity of the F-functionalized rim.

**Table 2:** Average hydrogen bond formation between water molecules and functionalized groups on the pore edge under different applied electric fields

Electric field, kcal mol^−1^ Å^−1^e^−1^	System	Species	Hydrogen bond	
			Hg^2+^ ion	Co^2+^ ion
5	F–GRA	Water–F	1.62	1.38
20			1.21	1.05
35			0.84	0.71
5	Cl–GRA	Water–Cl	0.94	1.08
20			0.72	0.86
35			0.51	0.63

The Cl-functionalized pores exhibit a contrasting interfacial behavior. Owing to the lower electronegativity, hydrogen bonding between water and the Cl-terminated rim is inherently weaker (Fig. 3b). Under equivalent field conditions, Co^2+^ exhibits a greater propensity to form water–Cl hydrogen bonds compared to Hg^2+^, arising from its stronger hydration shell stability and higher effective charge density, which improve the orientational structuring of water near the Cl-terminated pore. Across both pore chemistries, water molecules traversing the subnanometer aperture adopt a single-file arrangement imposed by spatial confinement, making the hydrogen-bonding environment highly sensitive to the interplay between pore functionalization, local electrostatics, and ion–solvent dynamics.

### 3.6 Interaction energy

To elucidate the energetic contributions governing selective ion permeation across the functionalized GRA pores, interaction energies between different species in the membrane separation system were computed using the NAMD Energy plugin. This plugin evaluates interaction energies between selected atom groups for each frame of the simulation trajectory. The final interaction energies were obtained by averaging over multiple trajectory frames, thereby minimizing statistical fluctuations and ensuring representative values. The total interaction energy was calculated as the sum of the electrostatic, van der Waals, and non-bonded interaction energies. As summarized in Table 3, all systems exhibit negative interaction energies, indicating favourable attractive interactions among the species. As the electric field increases, the driving force acting on the metal ions becomes stronger, pushing them closer to the pore rim and intensifying their interaction with the functionalized GRA surface. This field-induced confinement reduces the ion–surface distance and enhances electrostatic coupling, resulting in progressively more negative metal–GRA interaction energies in both F-pore and Cl-pore systems. Notably, the absolute interaction strength is higher in the F-pore due to its stronger local electronegativity. Among the ions, Co^2+^–GRA interaction energy (*i.e.*, −210.11 kcal mol^−1^) was found to be higher than that of Hg^2+^–GRA (*i.e.*, −149.02 kcal mol^−1^), indicating a stronger affinity of Co^2+^ toward the fluorinated nanoporous GRA membrane. This difference arises from their distinct physicochemical characteristics. The stronger Co^2+^–GRA interaction reduces the Co^2+^–water interaction (*i.e.*, −55.36 kcal mol^−1^), promoting partial dehydration and stabilizing the ion near the pore entrance. Under higher electric field strengths, this stabilization facilitates its subsequent permeation through the F–GRA membrane, consistent with the RDF trends shown in Fig. 5. With stronger field-driven forcing, the metal–water interaction energy decreases due to increased perturbation of the hydration shell, leading to gradual loss of coordinated water molecules during ion approach to the pore. This reduction is more pronounced in the F-pore system, where highly electronegative F atoms create stronger competing ion-pore interactions that accelerate hydration-shell disruption. In contrast, the Cl-pore exhibits weaker ion-pore coupling, allowing the ions to retain a larger portion of their hydration shell and resulting in a comparatively smaller reduction in metal–water interaction energy, further corroborating the dehydration behavior observed in Fig. 6.

**Table 3:** Interaction energy between different molecules

Electric field, kcal mol^−1^ Å^−1^ e^−1^	Species	Interaction energy (kcal mol^−1^)			
		F–GRA		Cl–GRA	
		Hg^2+^ ion	Co^2+^ ion	Hg^2+^ ion	Co^2+^ ion
5	Metal–GRA	−89.69	−137.20	−119.33	−77.01
	Water–GRA	−384.81		−280.45	
	Metal–water	−146.85	−112.38	−128.08	−143.22
20	Metal–GRA	−130.31	−197.25	−145.21	−111.33
	Water–GRA	−393.30		−311.77	
	Metal–water	−113.92	−61.35	−83.21	−100.30
35	Metal–GRA	−149.02	−210.11	−187.11	−138.21
	Water–GRA	−426.88		−355.08	
	Metal–water	−90.16	−55.36	−77.33	−89.71

Furthermore, the water–GRA interaction energy becomes more negative with increasing electric field strength, as the field induces stronger dipole alignment of water molecules and drives them closer to the pore surface, thereby enhancing interfacial interactions. This effect is particularly pronounced in the F-pore system (*i.e.*, −426.88 kcal mol^−1^). Overall, the interplay between metal ion–GRA, metal ion–water, and water–GRA interactions supported by both interaction energy analysis and RDF results provides a coherent mechanistic explanation for the observed ion selectivity during transport through F–GRA and Cl–GRA membranes.

### 3.7 Diffusion dynamics

**Fig. 9:**
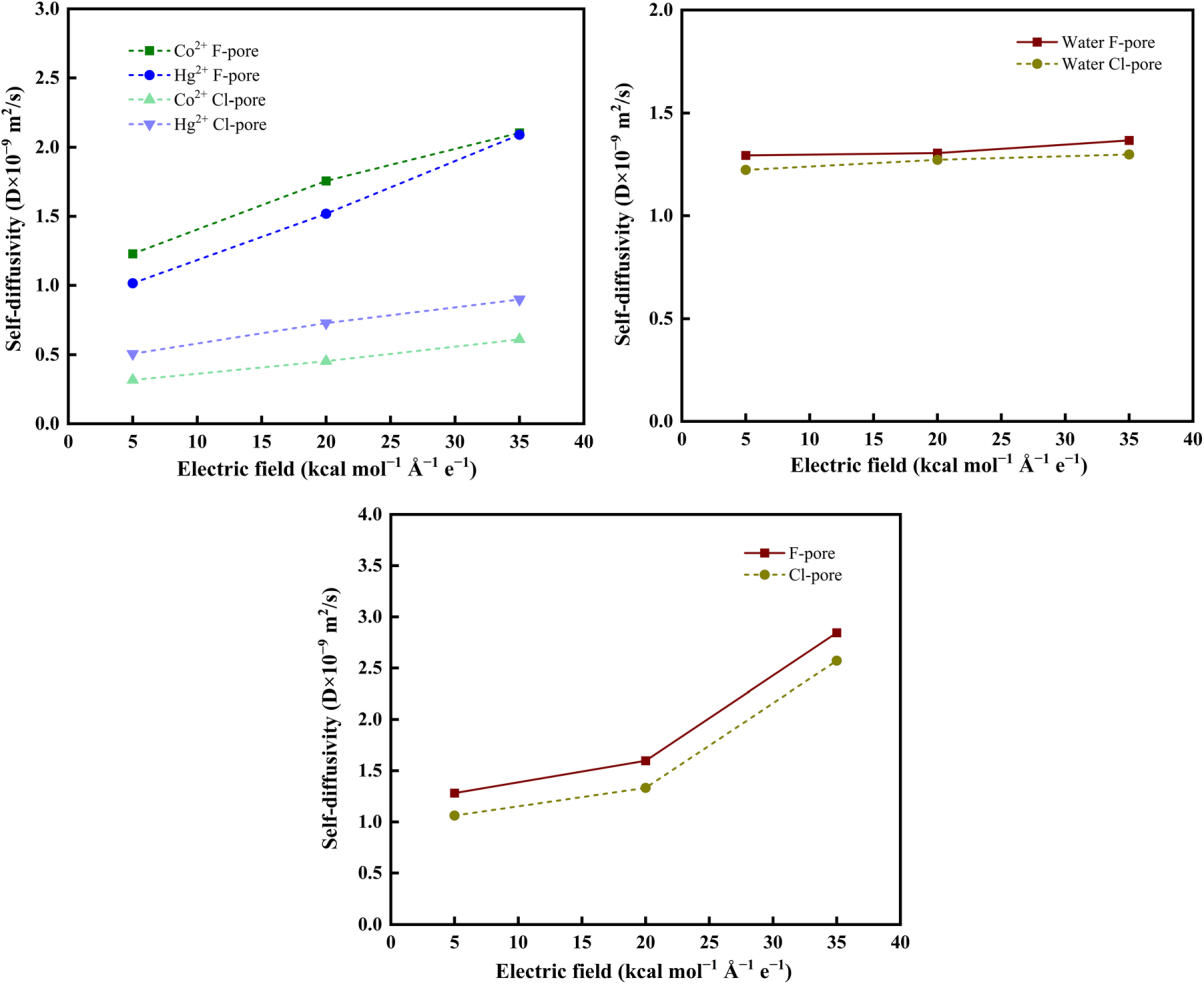
Self-diffusivity of (a) heavy metal ions, (b) water molecules, and (c) NO_3_^−^ ions in the F-pore and Cl-pore system.

The self-diffusion coefficients of metal ions and water molecules were evaluated in the vicinity of the nanopore using the mean square displacement (MSD) and the Einstein relation (see eqn (5)). This local analysis was performed to capture the mobility of species under nanoscale confinement during permeation. In the pore region, ions and water molecules experience strong interactions with the functionalized pore edges and undergo partial dehydration, which can significantly alter their dynamics compared to those in the bulk solution. Therefore, evaluating the diffusion coefficient in the pore vicinity provides a more representative description of the transport behavior governing permeation through the membrane. The self-diffusivity of heavy metal ions were depicted in Fig. 9a. In the fluorinated pore, Co^2+^ ions exhibit a slightly higher effective diffusivity (*i.e.*, 2.104 × 10^−9^ m^2^ s^−1^ at 35 kcal mol^−1^ Å^−1^ e^−1^) compared to Hg^2+^ (*i.e.*, 2.091 × 10^−9^ m^2^ s^−1^ at 35 kcal mol^−1^ Å^−1^ e^−1^). This difference arises from the combined influence of pore chemistry and the applied electric field. The high electronegativity of F atoms enhances electrostatic attraction toward the ions and promotes perturbation of their hydration shells. Moreover, the external electric field exerts a directional force on charged species, thereby facilitating their transport along the field direction. In contrast, the highly polarizable Hg^2+^ ion, with its lower charge density, interacts with the fluorinated pore edges primarily through van der Waals and polarization-driven forces, resulting in comparatively weaker directional transport.

On the other hand, Hg^2+^ ions exhibit slightly higher (*i.e.*, 0.900 × 10^−9^ m^2^ s^−1^ at 35 kcal mol^−1^ Å^−1^ e^−1^) mobility relative to Co^2+^ (*i.e.*, 0.611 × 10^−9^ m^2^ s^−1^ at 35 kcal mol^−1^ Å^−1^ e^−1^) due to the enhanced polarizability of Cl atoms at the pore rim, which favors stronger but more flexible interactions with the Hg^2+^ ions. As a result, Hg^2+^ can migrate more readily through the Cl-modified nanopore compared to the Co^2+^ ion. This diffusivity trend aligns well with the RDFs, hydrogen bonding, and interaction energy, reinforcing that the chemical nature of the pore edge critically influences ion–solvent coupling, hydration-shell restructuring, and ultimately the selective permeation behavior under an applied electric field.

The diffusion dynamics of water molecules exhibit a systematic increase with rising electric field strength in both the F-pore and Cl-pore systems (Fig. 9b). This enhancement in diffusivity arises from the field-induced acceleration of the electroosmotic flow, where the external electric field imposes a directional force on solvated ions and the surrounding hydration water. As ions migrate toward the nanopore, they generate a local advective drag that disrupts the hydrogen-bond network and enhances the translational mobility of neighbouring water molecules. Consequently, the confined solvent experiences faster rearrangements and reduced residence times, leading to higher self-diffusivity at elevated field strengths. Despite this, the water diffusivity remains consistently higher in the F-functionalized pore (*i.e.*, 1.367 × 10^−9^ m^2^ s^−1^ at 35 kcal mol^−1^ Å^−1^ e^−1^) relative to the Cl-functionalized pore (*i.e.*, 1.298 × 10^−9^ m^2^ s^−1^ at 35 kcal mol^−1^ Å^−1^ e^−1^). This behavior is attributed to enhanced water–surface interactions associated with the high electronegativity of fluorine under an external electric field, as evidenced by the interaction energy values in Table 3. Thus, the combined effects of electric field and pore-edge chemistry govern the overall water diffusivity. An increase in anion diffusivity (Fig. 9c) is observed in both pore systems with increasing external electric field strength. This trend aligns well with the dehydration behaviour (Fig. 6c) and the corresponding permeation characteristics (Fig. 8b), indicating a clear interplay between ion mobility, hydration dynamics, and transport behaviour.

## 4 Conclusions

One major area of interest in the field of water purification is the removal of heavy metals from water. In this research work, a classical MD simulation approach was utilized to investigate the selective permeation of heavy metal ions *via* two distinct (*i.e.*, fluorine and chlorine) functionalized nanoporous GRA structures. The membrane design effectively combined nanoscale confinement and electric field-induced selectivity to achieve efficient separation of Hg^2+^ and Co^2+^ from nitrate-rich wastewater. It has been observed that F-functionalized nanoporous GRA membranes can facilitate the overall ion permeation when subjected to an external electric field. Particularly, Co^2+^ ions show preferential permeation through the F-pore, whereas Hg^2+^ ions permeate more readily through the Cl-pore. The EOF technique is an effective approach for transferring analytes. In terms of energy efficiency, utilizing electric fields to drive ion transport can be more energy-efficient compared to traditional pressure-driven filtration methods. Moreover, the selective permeation of Hg^2+^ and Co^2+^ ions, coupled with the minimal passage of NO_3_^−^ ions, was highlighted in this investigation. The presence of NO_3_^−^ ions in the permeate side, despite the system's selectivity, can be attributed to high electric field strength, thermal fluctuations, and transient pore dynamics. Complete rejection of NO_3_^−^ ions is challenging, and minimal leakage does not necessarily indicate a failure of the system but rather reflects the complex interplay of forces at the nanoscale. The current study's results are expected to serve as a foundation for future research and as an initiative for creating energy-efficient GRA-based heavy metal separation instruments. Additionally, understanding the influence of pore chemistry can guide the development of next-generation membranes tailored for the selective removal of specific contaminants.

## Author contributions

Drisya G. Chandran: writing – original draft, writing – review & editing, methodology, investigation, formal analysis, visualization, data curation, conceptualization. Rima Biswas: writing – review & editing, supervision, methodology, investigation, formal analysis, visualization, data curation, conceptualization.

## Conflicts of interest

The authors declare that they have no known competing financial interests or personal relationships that could have appeared to influence the work reported in this paper.

## Supplementary Material

RA-016-D6RA00091F-s001

## Data Availability

The authors confirm that the data supporting the findings of this study are available within the article and its supplementary information (SI) file, which is provided separately. No additional source data are required. Supplementary information is available. See DOI: https://doi.org/10.1039/d6ra00091f.
