# Supplementary material for: Unravelling the selective transport of Co2+ and Hg2+ ions through functionalized graphene nanostructures from aqueous nitrate solution: a molecular dynamics simulation study
Source: RSC Adv. 2026 May 8;16(26):24040–54. doi: 10.1039/d6ra00091f (PMC13154393; doi:10.1039/d6ra00091f)
Supplement: RA-016-D6RA00091F-s001 [file RA-016-D6RA00091F-s001.pdf]

Supplementary Information (SI) for RSC Advances.
This journal is © The Royal Society of Chemistry 2026

Supplementary information (SI)

Unravelling the Selective Transport of Co^{2+} and Hg^{2+} Ions through Functionalized Graphene Nanostructures from Aqueous Nitrate Solution: A Molecular Dynamics Simulation Study

Drisya G. Chandran[a], Rima Biswas[*a]

[a] Process Simulation Research Group, School of Chemical Engineering, Vellore Institute of Technology, Vellore, Tamil Nadu, India 632014

*Corresponding author E-mail: rima.biswas@vit.ac.in

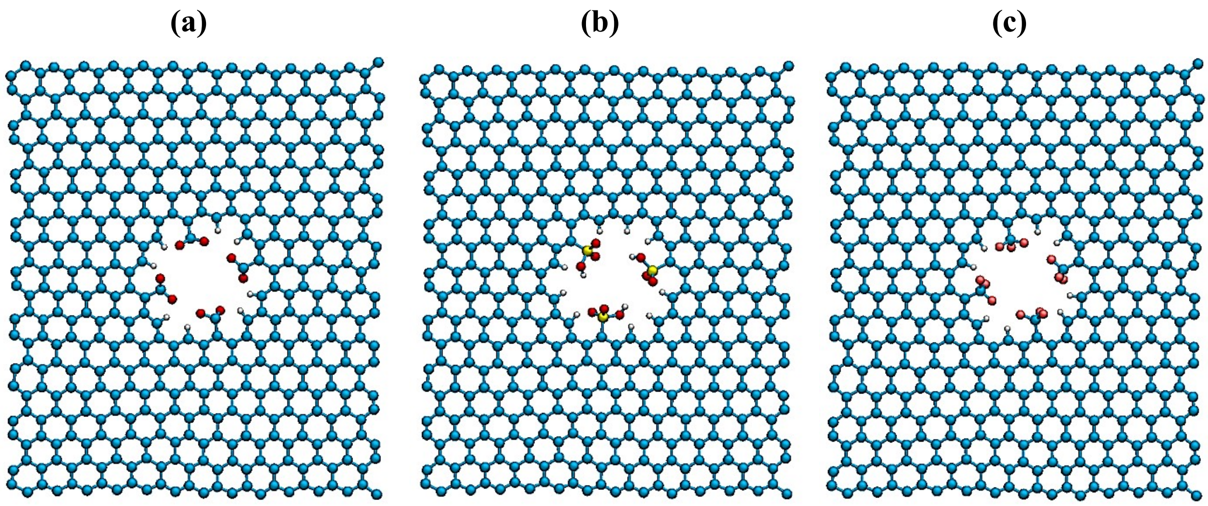

Fig. S1 Snapshots of nanoporous GRA membrane functionalized with (a) $–COO^-$, (b) $–SO_3H$, and (c) $–CF_3$. Carbon, oxygen, hydrogen, sulphur, and fluorine are represented by cyan, red, white, yellow, and pink colors respectively.

Table S1 List of non-bonding parameters

Molecules/Ions	Site	Charge (e)	σ(Å)	ε(kcal/mol)
COO^-–GRA	C(GRA)	0.1000	3.4000	0.0860
	C	0.7000	3.7500	0.1090
	O(C–O)	−1.0000	2.9600	0.2100
	O(C=O)	−0.8000	2.9600	0.2100
SO_3H–GRA	C(GRA)	−0.0134	3.5418	−0.0700
	S	1.4177	3.5418	−0.2500
	O(S=O)	−0.5636	2.9531	−0.1700
	O(S–O)	−0.7151	3.1127	−0.1700
	H	0.5316	0.0000	−0.0000
CF_3–GRA	C(GRA)	−0.2042	3.5418	−0.0700
	C	0.4966	3.4919	−0.0660
	F	−0.1685	2.8933	−0.0600
